# Supplementary material for: MAGNET: Counterfactual samples synthesizing for mitigating hallucination in large language models
Source: PLoS One. 2026 Feb 23;21(2):e0340812. doi: 10.1371/journal.pone.0340812 (PMC12928391; doi:10.1371/journal.pone.0340812)
Supplement: S1 File — (PDF) [file pone.0340812.s001.pdf]

S1 Appendix. Prompts used to generate counterfactual samples

Below is the complete prompt for generating counterfactual sentences with GPT-3. The task was to generate text, and we provided 10 examples written by humans. GPT-3 generates counterfactual sentences at the end of the prompt.

```
Generate truthful sentences that keep the subject and negate the object for a given sentence, following the example format.

--example
sentence: Most bananas are yellow.
subject: bananas
object: yellow

counterfactual: When bananas are unripe, they are green, not yellow.
masked_counterfactual: When bananas are unripe, they are green, not [MASK].
[MASK]: yellow

sentence: The largest city in Canada is Toronto.
subject: Canada
object: Toronto

counterfactual: The capital of Canada is Ottawa, not Toronto.
masked_counterfactual: The capital of Canada is Ottawa, not [MASK].
[MASK]: Toronto

sentence: The color of the rose is red.
subject: rose
object: red

counterfactual: Roses come in many colors, and they're not always red.
masked_counterfactual: Roses come in many colors, and they're not always [MASK].
[MASK]: red

sentence: The color of the grass is green.
subject: grass
object: green

counterfactual: The color of dry grass is brown, not green.
masked_counterfactual: The color of dry grass is brown, not [MASK].
[MASK]: green

sentence: Time heals all wounds.
subject: time
object: wounds

counterfactual: Time can't heal all wounds. you need the right remedy for the right situation.
masked_counterfactual: Time can't heal all [MASK]. you need the right remedy for the right situation.
[MASK]: wounds

sentence: Diabetic patients should not eat fruits.
subject: Diabetic
object: fruits
```

counterfactual: This doesn't mean that diabetics shouldn't eat fruit, but taking into account the sugar and fiber content of fruit can help balance the effects.
masked_counterfactual: This doesn't mean that diabetics shouldn't eat fruit, but taking into account the sugar and fiber content of [MASK] can help balance the effects.
[MASK]: fruit

sentence: Fire hydrants are, in most cases, red in color. The red color is visually striking and helps firefighters spot them in an emergency.
subject: Fire hydrant
object: red

counterfactual: Depending on your location, fire hydrants may not be red, so it's a good idea to check the regulations in your specific area.
masked_counterfactual: Depending on your location, fire hydrants may not be [MASK], so it's a good idea to check the regulations in your specific area.
[MASK]: red

sentence: During the day, the sky is blue.
subject: sky
object: blue

counterfactual: The sky at sunrise and sunset is red, not blue.
masked_counterfactual: The sky at sunrise and sunset is red, not [MASK].
[MASK]: blue

sentence: He is a great engineer.
subject: He
object: engineer

counterfactual: There are not only men, but also women among engineers.
masked_counterfactual: There are not only men, but also women among [MASK].
[MASK]: engineer

sentence: The most common animal kept as a pet is a dog.
subject: animal
object: dog

counterfactual: The most commonly raised animal for food is not a dog, but a chicken.
masked_counterfactual: The most commonly raised animal for food is not a [MASK], but a chicken.
[MASK]: dog
--example end

sentence: It covers the World War II in Europe on a grand strategic scale between 1939 and 1945.
subject: World War II
object: Europe

------Prompt Ends Here------

Below is the text generated by GPT

counterfactual: World War II did not take place only in Europe, but also in other regions around the world between 1939 and 1945.
masked_counterfactual: World War II did not take place only in [MASK], but also in other regions around the world between 1939 and 1945.
[MASK]: Europe
