# Supplementary material for: MAGNET: Counterfactual samples synthesizing for mitigating hallucination in large language models
Source: PLoS One. 2026 Feb 23;21(2):e0340812. doi: 10.1371/journal.pone.0340812 (PMC12928391; doi:10.1371/journal.pone.0340812)
Supplement: Attachment — Submitted filename: Response Paper.pdf [file pone.0340812.s004.pdf]

**Original Manuscript ID**	**PONE-D-25-17618**
**Original Article Title**	**MAGNET: Counterfactual samples synthesizing for mitigating hallucination in large language models**

**Reviewer #1 - Comment 1**	
**Comment**	Add statistical tests (like p-values or confidence intervals) to show that the results are reliable.
**Response**	We thank the reviewer for the valuable feedback regarding the statistical reliability of our results. In response, we have updated Tables 4, 5, 6, and 7 to include 95% confidence intervals (CI) for all reported metrics, computed over 5 independent runs. By providing these CIs, we quantify the variability in the results and demonstrate that the observed improvements with MAGNET and across different experimental settings are statistically reliable. The mean ± 95% CI format allows readers to assess the significance and robustness of the reported performance gains.<br><br>**[Page 10-11](Table 4, Table 5, Table 6, Table 7) – Section Results**
**Reviewer #1 - Comment 2**	
**Comment**	Do ablation studies to show how different prompts and data filtering affect the results.
**Response**	We sincerely thank the reviewer for the helpful suggestion. To address this, we conducted ablation experiments to evaluate how different prompt settings and human filtering affect MAGNET's performance. We compared 1-shot, 5-shot, and 10-shot prompts to investigate the impact of the number of in-context examples. Additionally, for the 10-shot setting, we applied human filtering to ensure that the generated counterfactual sentences preserved the subject-object pairs and were factually accurate.<br><br>The results indicate that increasing the number of shots improves performance, as the model can better follow the given examples. Furthermore, human filtering on the 10-shot setting consistently enhanced performance across all benchmarks, demonstrating the importance of maintaining factual correctness and the subject-object structure in counterfactual data for effective MAGNET fine-tuning.<br><br>**[Page 10-11](Line 299–323) – Section Ablation Study of Results**<br>**Ablation Study**<br>For assessing the quality of counterfactual sentences, we conducted generation 300 experiments with 1-shot, 5-shot, and 10-shot prompt settings.<br><br>Table 5 shows that as the number of shots increases, the model is better able to follow the given prompt and produce correctly formatted outputs, which allows for more effective data collection. Across the different n-shot settings, sentences generated with 10-shot prompts led to the best performance.<br><br>We conducted human filtering to ensure that the generated sentences preserved the subject and object while remaining factually consistent.<br><br>Table 6 shows that human filtering was performed to ensure that generated sentences preserved the subject and object and were factually accurate, which improved performance across all benchmarks.<br><br>Together with the n-shot setting, these results indicate that maintaining the subject and object and generating factually grounded sentences, as proposed in this study, contributes to higher model performance.<br><br>Table 7 summarizes the performance of MAGNET when using different LLMs for counterfactual generation. While our experiments primarily used the Turbo models, as is common in many practical settings, GPT-4 demonstrated improved performance across all benchmarks compared to GPT-3.5. This highlights the importance of high-quality counterfactual samples for effective

MAGNET fine-tuning. Furthermore, it emphasizes that generating factually grounded sentences while preserving the subject-object structure, employing a sufficient number of in-context shots, and leveraging up-to-date, high-capacity language models are key factors for maximizing MAGNET's effectiveness. These findings suggest that adopting newer models in future applications can further enhance the factual robustness and truthfulness of LLMs.

Reviewer #1 - Comment 3

Comment Look into cases where the method makes correct answers wrong, and explain why that happens.

Response We thank the reviewer for this insightful comment. To address this, we analyzed instances where MAGNET flipped originally correct predictions into incorrect ones in the Factual Knowledge Probing experiments. As shown in Table 2 and Table 3, these cases mainly occur when the distribution of possible objects for a given subject is relatively uniform—i.e., no single object strongly dominates. In such situations, even though the ground truth has a higher conditional probability, MAGNET occasionally changes the prediction due to the introduction of counterfactual samples. This highlights a trade-off of counterfactual training: while it improves robustness against spurious co-occurrence biases, it can slightly affect predictions when multiple plausible objects exist for a subject.

[Page 8-9](Line 262–280) – Section Correlation Analysis of Results

Correlation Analysis

Without MAGNET, GPT-Neo 2.7B produced 5,154 correct and 3,670 incorrect answers out of 8,824. With MAGNET, the model achieved 6,177 correct and 2,647 incorrect answers, meaning that 1,521 cases were changed from incorrect to correct, while 498 cases were changed from correct to incorrect.

Among the 3,670 errors without MAGNET, we identified 989 cases where the model predicted a word with a higher co-occurrence count than the ground truth. Of these bias-induced errors, 311 were corrected by MAGNET (examples in Table 2). Conversely, among the 498 answers that changed from correct to incorrect, 382 had higher conditional probability on the ground truth under the base model, indicating that MAGNET occasionally flipped answers despite the original model preferring the correct option (examples in Table 3).

Our analysis confirms that MAGNET effectively corrects bias-induced errors while occasionally flipping originally correct answers to incorrect ones. We observed that these "correct → incorrect" cases tend to occur when the distribution of potential objects for a given subject is relatively uniform, i.e., no single object dominates the co-occurrence statistics. As a future direction, we hypothesize that constraining counterfactual sentence generation to subjects whose objects exhibit a strong skew (above a certain threshold) could reduce unnecessary flipping and further improve overall model performance.

Reviewer #1 - Comment 4

Comment Fix grammar issues and make the writing clearer; also, make the tables easier to read.

Response We thank the reviewer for the suggestion. We have revised the manuscript to improve grammar and overall clarity. In addition, to enhance readability, Table 2—where readability was previously limited—has been replaced with Figure 6, which presents the information more clearly. Other tables have also been reformatted to improve accessibility and consistency.

[Page 8](Figure 6) – Section Correlation Analysis of Results

[Page 8-9](Line 262–280) – Section Correlation Analysis of Results

Correlation Analysis

Without MAGNET, GPT-Neo 2.7B produced 5,154 correct and 3,670 incorrect answers out of 8,824. With MAGNET, the model achieved 6,177 correct and 2,647 incorrect answers, meaning that 1,521 cases were changed from incorrect to correct, while 498 cases were changed from correct to incorrect.

Among the 3,670 errors without MAGNET, we identified 989 cases where the model predicted a word with a higher co-occurrence count than the ground truth. Of these bias-induced errors, 311 were corrected by MAGNET (examples in Table 2). Conversely, among the 498 answers that changed from correct to incorrect, 382 had higher conditional probability on the ground truth under the base model, indicating that MAGNET occasionally flipped answers despite the original model preferring the correct option (examples in Table 3).

	Our analysis confirms that MAGNET effectively corrects bias-induced errors while occasionally flipping originally correct answers to incorrect ones. We observed that these "correct → incorrect" cases tend to occur when the distribution of potential objects for a given subject is relatively uniform, i.e., no single object dominates the co-occurrence statistics. As a future direction, we hypothesize that constraining counterfactual sentence generation to subjects whose objects exhibit a strong skew (above a certain threshold) could reduce unnecessary flipping and further improve overall model performance.<br>**[Page 9](Figure 9)**<br>Correlation between GPT-Neo 125M pre-training co-occurrence frequencies and Hits@1 scores with MAGNET, under the remove stopwords setting.
**Reviewer #1 - Comment 5**	
**Comment**	Share the exact prompts and settings used to generate the counterfactual data so others can repeat the work.
**Response**	We thank the reviewer for the suggestion. The exact prompts and generation settings used for creating the counterfactual data are provided in S1 Appendix, allowing other researchers to reproduce our experiments.<br><br>**[Page 13](Line 402) – S1 Appendix**<br>**S1 Appendix. Prompts used to generate counterfactual samples**<br><br>Below is the complete prompt for generating counterfactual sentences with GPT-3. The task was to generate text, and we provided 10 examples written by humans. GPT-3 generates counterfactual sentences at the end of the prompt.<br><br>Generate truthful sentences that keep the subject and negate the object for a given sentence, following the example format.<br><br>--example<br>sentence: Most bananas are yellow.<br>subject: bananas<br>object: yellow<br><br>counterfactual: When bananas are unripe, they are green, not yellow.<br>masked_counterfactual: When bananas are unripe, they are green, not [MASK].<br>[MASK]: yellow<br><br>sentence: The largest city in Canada is Toronto.<br>subject: Canada<br>object: Toronto<br><br>counterfactual: The capital of Canada is Ottawa, not Toronto.<br>masked_counterfactual: The capital of Canada is Ottawa, not [MASK].<br>[MASK]: Toronto<br><br>sentence: The color of the rose is red.<br>subject: rose<br>object: red<br><br>counterfactual: Roses come in many colors, and they're not always red.<br>masked_counterfactual: Roses come in many colors, and they're not always [MASK].<br>[MASK]: red<br><br>sentence: The color of the grass is green.<br>subject: grass<br>object: green<br><br>counterfactual: The color of dry grass is brown, not green.<br>masked_counterfactual: The color of dry grass is brown, not [MASK].<br>[MASK]: green

sentence: Time heals all wounds.
subject: time
object: wounds

counterfactual: Time can't heal all wounds. you need the right remedy for the right situation.
masked_counterfactual: Time can't heal all [MASK]. you need the right remedy for the right situation.
[MASK]: wounds

sentence: Diabetic patients should not eat fruits.
subject: Diabetic
object: fruits

counterfactual: This doesn't mean that diabetics shouldn't eat fruit, but taking into account the sugar and fiber content of fruit can help balance the effects.
masked_counterfactual: This doesn't mean that diabetics shouldn't eat fruit, but taking into account the sugar and fiber content of [MASK] can help balance the effects.
[MASK]: fruit

sentence: Fire hydrants are, in most cases, red in color. The red color is visually striking and helps firefighters spot them in an emergency.
subject: Fire hydrant
object: red

counterfactual: Depending on your location, fire hydrants may not be red, so it's a good idea to check the regulations in your specific area.
masked_counterfactual: Depending on your location, fire hydrants may not be [MASK], so it's a good idea to check the regulations in your specific area.
[MASK]: red

sentence: During the day, the sky is blue.
subject: sky
object: blue

counterfactual: The sky at sunrise and sunset is red, not blue.
masked_counterfactual: The sky at sunrise and sunset is red, not [MASK].
[MASK]: blue

sentence: He is a great engineer.
subject: He
object: engineer

counterfactual: There are not only men, but also women among engineers.
masked_counterfactual: There are not only men, but also women among [MASK].
[MASK]: engineer

sentence: The most common animal kept as a pet is a dog.
subject: animal
object: dog

counterfactual: The most commonly raised animal for food is not a dog, but a chicken.
masked_counterfactual: The most commonly raised animal for food is not a [MASK], but a chicken.
[MASK]: dog
--example end

sentence: It covers the World War II in Europe on a grand strategic scale between 1939 and 1945.
subject: World War II
object: Europe

<table>
<tr><td></td><td>------Prompt Ends Here------

Below is the text generated by GPT

counterfactual: World War II did not take place only in Europe, but also in other regions around the world between 1939 and 1945.
masked_counterfactual: World War II did not take place only in [MASK], but also in other regions around the world between 1939 and 1945.
[MASK]: Europe</td></tr>
<tr><td colspan="2">Reviewer #2 - Comment 1</td></tr>
<tr><td>Comment</td><td>Include ablation experiments to quantify the contribution of each component (extraction, generation, filtering).</td></tr>
<tr><td>Response</td><td>We thank the reviewer for the suggestion. We conducted ablation experiments to assess the contribution of each component in MAGNET: prompt shot count (1-shot, 5-shot, 10-shot), human filtering (applied to 10-shot), and the counterfactual generation model (GPT-3.5 Turbo vs. GPT-4 Turbo).

[Page 10-11](Line 299-323) – Section Ablation Study of Results
Ablation Study
For assessing the quality of counterfactual sentences, we conducted generation 300 experiments with 1-shot, 5-shot, and 10-shot prompt settings.

Table 5 shows that as the number of shots increases, the model is better able to follow the given prompt and produce correctly formatted outputs, which allows for more effective data collection. Across the different n-shot settings, sentences generated with 10-shot prompts led to the best performance.

We conducted human filtering to ensure that the generated sentences preserved the subject and object while remaining factually consistent.

Table 6 shows that human filtering was performed to ensure that generated sentences preserved the subject and object and were factually accurate, which improved performance across all benchmarks.

Together with the n-shot setting, these results indicate that maintaining the subject and object and generating factually grounded sentences, as proposed in this study, contributes to higher model performance.

Table 7 summarizes the performance of MAGNET when using different LLMs for counterfactual generation. While our experiments primarily used the Turbo models, as is common in many practical settings, GPT-4 demonstrated improved performance across all benchmarks compared to GPT-3.5. This highlights the importance of high-quality counterfactual samples for effective MAGNET fine-tuning. Furthermore, it emphasizes that generating factually grounded sentences while preserving the subject-object structure, employing a sufficient number of in-context shots, and leveraging up-to-date, high-capacity language models are key factors for maximizing MAGNET's effectiveness. These findings suggest that adopting newer models in future applications can further enhance the factual robustness and truthfulness of LLMs.</td></tr>
<tr><td colspan="2">Reviewer #2 - Comment 2</td></tr>
<tr><td>Comment</td><td>Conduct statistical significance testing to confirm robustness of improvements.</td></tr>
<tr><td>Response</td><td>We thank the reviewer for the valuable feedback regarding the statistical reliability of our results. In response, we have updated Tables 4, 5, 6, and 7 to include 95% confidence intervals (CI) for all reported metrics, computed over 5 independent runs. By providing these CIs, we quantify the variability in the results and demonstrate that the observed improvements with MAGNET and across different experimental settings are statistically reliable. The mean ± 95% CI format allows readers to assess the significance and robustness of the reported performance gains.

[Page 10-11](Table 4, Table 5, Table 6, Table 7) – Section Results</td></tr>
<tr><td colspan="2">Reviewer #2 - Comment 3</td></tr>
</table>

**Comment**	Extend evaluation to additional open-domain QA or fact-checking datasets.
**Response**	We appreciate the suggestion. In addition to Factual Knowledge Probing, we evaluated MAGNET on open-domain and reasoning benchmarks, specifically HellaSwag (10-shot) and Winogrande (5-shot). These results, reported in Tables 4, 5, 6, and 7, show consistent improvements across different LLMs and confirm that MAGNET's counterfactual fine-tuning benefits both factual knowledge and commonsense reasoning tasks.<br><br>**[Page 9-11](Line 281-323) – Section Results**<br>**Results for Open LLM**<br>We describe the evaluation results obtained by applying MAGNET to the target model. In addition to TruthfulQA, we also evaluate on HellaSwag and Winogrande, and the scores are shown in Table 4. For TruthfulQA, we use MC2 (Multi-true), which is the normalized total probability assigned to the correct answer set given a question and multiple true/false answers. For HellaSwag, we use multiple-choice accuracy, which measures the proportion of cases where the model selects the correct continuation among four candidates. For Winogrande, we also use multiple-choice accuracy, defined as the percentage of correctly resolved pronoun disambiguation questions.<br><br>We trained the model on 4 RTX 3090 GPUs with 3 epochs and used a batch size of 256. The learning rate is 2e-5 and the optimizer is Adam with $\beta_1 = 0.9, \beta_2 = 0.999$. Everything else is the same as the script available in HuggingFace's causal language modeling [58]. The details of fine-tuning and testing are shown in S3 Table.<br><br>Here, experiments with adjusting the number of training data for GPT-Neo 125M are shown in Fig 9, and all experiments are just single runs.<br><br>Consequently, MAGNET eliminates the co-occurrence bias, reducing the probability of generating incorrect words that predict high co-occurrence, which benefits the truthfulness of the sentence.<br><br>**Ablation Study**<br>For assessing the quality of counterfactual sentences, we conducted generation 300 experiments with 1-shot, 5-shot, and 10-shot prompt settings.<br><br>Table 5 shows that as the number of shots increases, the model is better able to follow the given prompt and produce correctly formatted outputs, which allows for more effective data collection. Across the different n-shot settings, sentences generated with 10-shot prompts led to the best performance.<br><br>We conducted human filtering to ensure that the generated sentences preserved the subject and object while remaining factually consistent.<br><br>Table 6 shows that human filtering was performed to ensure that generated sentences preserved the subject and object and were factually accurate, which improved performance across all benchmarks.<br><br>Together with the n-shot setting, these results indicate that maintaining the subject and object and generating factually grounded sentences, as proposed in this study, contributes to higher model performance.<br><br>Table 7 summarizes the performance of MAGNET when using different LLMs for counterfactual generation. While our experiments primarily used the Turbo models, as is common in many practical settings, GPT-4 demonstrated improved performance across all benchmarks compared to GPT-3.5. This highlights the importance of high-quality counterfactual samples for effective MAGNET fine-tuning. Furthermore, it emphasizes that generating factually grounded sentences while preserving the subject-object structure, employing a sufficient number of in-context shots, and leveraging up-to-date, high-capacity language models are key factors for maximizing MAGNET's effectiveness. These findings suggest that adopting newer models in future applications can further enhance the factual robustness and truthfulness of LLMs.<br><br>58. Wolf T, Debut L, Sanh V, Chaumond J, Delangue C, Moi A, et al. Transformers: State-of-the-

	art natural language processing. In: Proceedings of the 2020 conference on empirical methods in natural language processing: system demonstrations; 2020. p. 38–45.
**Reviewer #2 - Comment 4**	
**Comment**	Discuss applicability to multi-hop reasoning and temporally sensitive facts.
**Response**	We thank the reviewer for this suggestion. We have added a discussion on this in the Discussion section. MAGNET primarily addresses single-hop factual associations by generating counterfactual samples to reduce co-occurrence biases. For multi-hop reasoning or temporally sensitive facts, the effectiveness may depend on the model's ability to maintain context across multiple reasoning steps or temporal updates. We note that approaches such as chain-of-thought prompting or retrieval-augmented generation (RAG) could complement MAGNET for these tasks, and we have highlighted this as a potential future direction.<br><br>**[Page 12](Line 362-370) – Section Discussion**<br>Furthermore, extending MAGNET to more complex reasoning settings remains an important avenue for future work. For instance, multi-hop reasoning often requires chaining intermediate facts, where co-occurrence biases can propagate across steps. Integrating MAGNET with chain-of-thought prompting may help stabilize such reasoning by reducing spurious associations at each step. Similarly, in temporally sensitive tasks where factual correctness depends on evolving knowledge, combining MAGNET with retrieval-augmented generation (RAG) could ensure that counterfactual training remains aligned with up-to-date evidence. Together, these directions highlight the broader applicability of MAGNET beyond single-hop factual recall.
**Reviewer #2 - Comment 5**	
**Comment**	Assess linguistic quality and diversity of counterfactuals to ensure naturalness.
**Response**	We thank the reviewer for this suggestion. To assess the linguistic quality and diversity of the generated counterfactual sentences, we computed Self-BLEU, which measures the similarity among generated sentences. The counterfactuals achieved a Self-BLEU score of 0.4668, indicating moderate diversity. This suggests that the generated sentences are sufficiently varied while maintaining naturalness and coherence, which is crucial for effective fine-tuning of the target model.<br><br>**[Page 6](Line 206-210) – Section Factual Knowledge Probing of Results**<br>To further assess the quality of the generated counterfactual samples, we computed Self-BLEU, a metric that measures the similarity between sentences. The counterfactual sentences achieved a Self-BLEU score of 0.4668, indicating moderate diversity. This suggests that the generated sentences are sufficiently varied while maintaining naturalness and coherence, which is crucial for effective fine-tuning of the target model.
**Reviewer #2 - Comment 6**	
**Comment**	Explore confidence-based weighting of counterfactuals to reduce overcorrection.
**Response**	We thank the reviewer for the suggestion. In our analysis, we confirmed that MAGNET effectively mitigates bias-induced errors, but a small portion of originally correct predictions were occasionally flipped to incorrect ones. These cases typically arise when the distribution of possible objects for a given subject is relatively uniform. As a future direction, we propose that constraining counterfactual generation to subjects whose objects exhibit a strong skew (above a certain threshold) could reduce unnecessary flips and improve overall model performance. This aligns with the idea of confidence-based weighting of counterfactuals, which could dynamically prioritize more informative samples.<br><br>**[Page 8-9](Line 262–280) – Section Correlation Analysis of Results**<br>**Correlation Analysis**<br>Without MAGNET, GPT-Neo 2.7B produced 5,154 correct and 3,670 incorrect answers out of 8,824. With MAGNET, the model achieved 6,177 correct and 2,647 incorrect answers, meaning that 1,521 cases were changed from incorrect to correct, while 498 cases were changed from correct to incorrect.<br><br>Among the 3,670 errors without MAGNET, we identified 989 cases where the model predicted a word with a higher co-occurrence count than the ground truth. Of these bias-induced errors, 311 were corrected by MAGNET (examples in Table 2). Conversely, among the 498 answers that changed from correct to incorrect, 382 had higher conditional probability on the ground truth

	under the base model, indicating that MAGNET occasionally flipped answers despite the original model preferring the correct option (examples in Table 3).<br><br>Our analysis confirms that MAGNET effectively corrects bias-induced errors while occasionally flipping originally correct answers to incorrect ones. We observed that these “correct → incorrect” cases tend to occur when the distribution of potential objects for a given subject is relatively uniform, i.e., no single object dominates the co-occurrence statistics. As a future direction, we hypothesize that constraining counterfactual sentence generation to subjects whose objects exhibit a strong skew (above a certain threshold) could reduce unnecessary flipping and further improve overall model performance.

Reviewer #3 - Comment 1	
**Comment**	Circular contamination problem – the authors are using GPT-3 to generate counterfactual samples, but GPT-3 itself hallucinates, so essentially it's like the models are trained on hallucinated "corrections." To be more specific, it MAGNET wants to negate "blue" in the figure 1 example, the underlying LLM needs to know when the sky is not blue, which also requires it's knowledge to be correct.
**Response**	We appreciate the reviewer's insightful comment regarding the potential circular contamination when using GPT-3 to generate counterfactual samples. In response, we conducted human filtering of the generated counterfactual sentences and confirmed that it is crucial to filter out hallucinated sentences to ensure that the generated data is factually grounded and preserves the subject-object structure. This step validated that MAGNET's performance benefits from high-quality counterfactuals. Additionally, we discuss in the manuscript that leveraging approaches such as Chain-of-Thought (CoT) prompting or Retrieval-Augmented Generation (RAG) in future work could further improve the LLM's knowledge, potentially reducing reliance on human filtering while maintaining the factual accuracy of generated samples.<br><br>**[Page 10-11](Line 308-310, Table 6) – Section Ablation Study of Results**<br>Table 6 shows that human filtering was performed to ensure that generated sentences preserved the subject and object and were factually accurate, which improved performance across all benchmarks.<br><br>**[Page 12](Line 362-370) – Section Discussion**<br>Furthermore, extending MAGNET to more complex reasoning settings remains an important avenue for future work. For instance, multi-hop reasoning often requires chaining intermediate facts, where co-occurrence biases can propagate across steps. Integrating MAGNET with chain-of-thought prompting may help stabilize such reasoning by reducing spurious associations at each step. Similarly, in temporally sensitive tasks where factual correctness depends on evolving knowledge, combining MAGNET with retrieval-augmented generation (RAG) could ensure that counterfactual training remains aligned with up-to-date evidence. Together, these directions highlight the broader applicability of MAGNET beyond single-hop factual recall.
**Reviewer #3 - Comment 2**	
**Comment**	The choice of GPT-3 for counterfactual generation seems outdated for a 2025 submission - GPT-4 or newer models would be more relevant for practical applications.
**Response**	We thank the reviewer for this suggestion. While our main experiments used GPT-3.5 Turbo due to common practical considerations, we additionally conducted experiments using GPT-4 Turbo for counterfactual generation. As shown in Table 7, GPT-4 Turbo yielded improved performance across all benchmarks (TruthfulQA, HellaSwag, and Winogrande) compared to GPT-3.5 Turbo. This demonstrates that MAGNET's effectiveness benefits from using higher-capacity, more up-to-date language models for generating counterfactual samples, confirming the relevance of employing GPT-4 or newer models in practical applications.<br><br>**[Page 11](Line 314–323, Table 7) – Section Ablation Study of Results**<br>Table 7 summarizes the performance of MAGNET when using different LLMs for counterfactual generation. While our experiments primarily used the Turbo models, as is common in many practical settings, GPT-4 demonstrated improved performance across all benchmarks compared to GPT-3.5. This highlights the importance of high-quality counterfactual samples for effective MAGNET fine-tuning. Furthermore, it emphasizes that generating factually grounded sentences while preserving the subject-object structure, employing a sufficient number of in-context shots, and leveraging up-to-date, high-capacity language models are key factors for maximizing MAGNET's effectiveness. These findings suggest that adopting newer models in future applications can further enhance the factual robustness and truthfulness of LLMs.
**Reviewer #3 - Comment 3**	
**Comment**	There is also no comparison to other finetuning methods that address hallucinations (eg. Ref [10]) at all.
**Response**	We thank the reviewer for the comment. In our study, we did not use the exact same metrics as Ref [10], and the prior work does not provide detailed information on how undersampling was conducted. To provide a fair comparison, we implemented an undersampling approach aligned with our experimental setup and evaluated it using our metrics. The results are reported in Table

	1, showing that undersampling reduces generalization performance, particularly in settings requiring a broad understanding of subject-object pairs. In contrast, MAGNET achieves superior performance by generating counterfactual samples rather than removing data, highlighting the advantage of our approach in addressing hallucination-related biases.<br><br>**[Page 7](Line 244–236, Table 1) – Section Factual Knowledge Probing of Results**<br>Table 1 presents the performance comparison of GPT-Neo 2.7B on the Factual Knowledge Probing task under different training strategies. The Baseline model is fine-tuned without considering subject-object co-occurrence biases, resulting in moderate Hits@1 scores across all evaluation settings. The Undersampling approach attempts to mitigate these biases by removing samples with high co-occurrence frequencies, but this reduces the overall training data. Consequently, the model is exposed to fewer diverse examples, increasing the risk of overfitting and impairing generalization, which is particularly evident in the Gold Objects and Relation-wise evaluation settings. In contrast, MAGNET addresses the co-occurrence bias by generating counterfactual samples that negate frequent object associations while preserving subjects. This allows the model to learn from both original and counterfactual data, maintaining diversity and improving generalization, resulting in substantially higher Hits@1 scores across all evaluation scenarios.
**Reviewer #3 - Comment 4**	
**Comment**	In the reported numbers more carefully, MAGNET corrected 311 incorrect predictions but caused 498 new errors from previously correct answers - a net loss of 187 correct responses. The claimed 12% improvement needs clearer explanation given this apparent contradiction.
**Response**	We thank the reviewer for the observation. The original description of MAGNET's net effect on correct and incorrect predictions may have been confusing. We have revised the wording to clarify the results.<br><br>**[Page 8-9](Line 262–280) – Section Correlation Analysis of Results**<br>**Correlation Analysis**<br>Without MAGNET, GPT-Neo 2.7B produced 5,154 correct and 3,670 incorrect answers out of 8,824. With MAGNET, the model achieved 6,177 correct and 2,647 incorrect answers, meaning that 1,521 cases were changed from incorrect to correct, while 498 cases were changed from correct to incorrect.<br><br>Among the 3,670 errors without MAGNET, we identified 989 cases where the model predicted a word with a higher co-occurrence count than the ground truth. Of these bias-induced errors, 311 were corrected by MAGNET (examples in Table 2). Conversely, among the 498 answers that changed from correct to incorrect, 382 had higher conditional probability on the ground truth under the base model, indicating that MAGNET occasionally flipped answers despite the original model preferring the correct option (examples in Table 3).<br><br>Our analysis confirms that MAGNET effectively corrects bias-induced errors while occasionally flipping originally correct answers to incorrect ones. We observed that these "correct → incorrect" cases tend to occur when the distribution of potential objects for a given subject is relatively uniform, i.e., no single object dominates the co-occurrence statistics. As a future direction, we hypothesize that constraining counterfactual sentence generation to subjects whose objects exhibit a strong skew (above a certain threshold) could reduce unnecessary flipping and further improve overall model performance.
**Reviewer #3 - Comment 5**	
**Comment**	Some presentation issues detract from the work: the figures are difficult to read due to low resolution, the lengthy tables interrupt the narrative flow (perhaps better suited for appendices), and Figure 7 would be more informative if it directly showed Hits@1 improvements rather than raw scores.
**Response**	We thank the reviewer for the helpful feedback. We have improved the resolution of all figures, moved lengthy tables to the appendices to enhance the flow of the main text, and clarified that the direct visualization of Hits@1 improvements, which relates to Figure 7, is provided in Figure 4.<br><br>**[Page 8-9](Line 262–280) – Section Correlation Analysis of Results**<br>**Correlation Analysis**<br>Without MAGNET, GPT-Neo 2.7B produced 5,154 correct and 3,670 incorrect answers out of

	8,824. With MAGNET, the model achieved 6,177 correct and 2,647 incorrect answers, meaning that 1,521 cases were changed from incorrect to correct, while 498 cases were changed from correct to incorrect.<br><br>Among the 3,670 errors without MAGNET, we identified 989 cases where the model predicted a word with a higher co-occurrence count than the ground truth. Of these bias-induced errors, 311 were corrected by MAGNET (examples in Table 2). Conversely, among the 498 answers that changed from correct to incorrect, 382 had higher conditional probability on the ground truth under the base model, indicating that MAGNET occasionally flipped answers despite the original model preferring the correct option (examples in Table 3).<br><br>Our analysis confirms that MAGNET effectively corrects bias-induced errors while occasionally flipping originally correct answers to incorrect ones. We observed that these “correct → incorrect” cases tend to occur when the distribution of potential objects for a given subject is relatively uniform, i.e., no single object dominates the co-occurrence statistics. As a future direction, we hypothesize that constraining counterfactual sentence generation to subjects whose objects exhibit a strong skew (above a certain threshold) could reduce unnecessary flipping and further improve overall model performance.<br>**[Page 9](Figure 9)**<br>Correlation between GPT-Neo 125M pre-training co-occurrence frequencies and Hits@1 scores with MAGNET, under the remove stopwords setting.<br>**[Page 13](Line 403-404) – S2 Table, S3 Table**
