# Supplementary material for: MAGNET: Counterfactual samples synthesizing for mitigating hallucination in large language models
Source: PLoS One. 2026 Feb 23;21(2):e0340812. doi: 10.1371/journal.pone.0340812 (PMC12928391; doi:10.1371/journal.pone.0340812)
Supplement: Attachment — Submitted filename: Response_Paper_auresp_2.pdf [file pone.0340812.s005.pdf]

| Original Manuscript ID | PONE-D-25-17618R1 |
|---|---|
| **Original Article Title** | **MAGNET: Counterfactual samples synthesizing for mitigating hallucination in large language models** |

| Reviewer #1 - Comment 1 | |
|---|---|
| **Comment** | Please ensure consistent formatting of performance metrics — for example, use "Hits@1" uniformly throughout the text, tables, and figures. |
| **Response** | We thank the reviewer for pointing out the inconsistency in the formatting of performance metrics. We have corrected the instance of "Hit@1" on line 212 to "Hits@1" and updated the caption of Figure 9, which was previously mislabeled. Consequently, the Results section now consistently uses the Hits@1 metric for Factual Knowledge Probing and Correlation Analysis, while the Results for Open LLM and Ablation Study sections report only the MC2 (Multi-true) and multiple-choice metrics, ensuring uniformity across the text, tables, and figure captions.<br><br>**[Page 6-11](Line 181-297) – Section Results**<br>**Results**<br><br>Table 1 provides a concise overview of the datasets, benchmarks, and evaluation metrics used in our experiments. Each experiment category—Factual Knowledge Probing, Counterfactual Training (MAGNET), Bias Analysis, and General Evaluation—is associated with its respective dataset and metrics, offering a clear summary of the experimental configuration prior to discussing detailed results.<br><br>**Factual Knowledge Probing**<br><br>Fig 4 shows the results of the Factual Knowledge Probing experiment in the study by \cite{kang2023impact}, which investigates the factual knowledge of LLMs using the LAMA-TREx dataset. The sentences used for validation are represented as subject-relation-object triples and converted into natural language using a predefined template. For example, the triple `Texas'-'capital'-'Austin' is converted to "The capital of Texas is Austin." Each fact masks the object and is converted into a Cloze statement (e.g., "The capital of Texas is [MASK]").<br><br>We trained the model for 3 epochs on 4 RTX 3090 GPUs. The batch size per device was 32, giving a total batch size of 128. The learning rate was 2e-5, and the Adam optimizer was used with beta_1 = 0.9 and beta_2 = 0.999.<br><br>For fine-tuning, the input prompt follows the format "### Input:\n {X\} \n\ n### Response:", where X is a masked sentence. For instance, "Hydatius has the position of [MASK]." The model is supervised to predict "bishop," which is the expected answer. Details are provided in S2 File.<br><br>The factual knowledge dataset contains 20,587 samples. We used 10,294 original sentences and 10,294 counterfactual samples as random samples to train MAGNET. To evaluate the quality of counterfactuals, we computed Self-BLEU, which measures sentence similarity. The score of 0.4668 indicates moderate diversity, showing that the generated sentences are sufficiently varied while remaining natural and coherent. This balance is important for effective fine-tuning.<br><br>For evaluation, we used Hits@1. It is 1 if the correct answer is ranked first among predicted candidates, and 0 otherwise. Because LLMs are not specifically trained for factual knowledge probing, we tested three restricted output vocabularies: (1) remove stopwords, (2) gold objects, and (3) gold objects (relation-wise). The first excludes NLTK 3.8.1 stopwords. The second restricts candidates to gold objects in the entire dataset, while the third restricts them per relation.<br><br>Fig 5 shows Hits@1 under a zero-shot setting with limited candidate sets. MAGNET improves |

| | |
|---|---|
| | the score by ~0.12 for the largest model and by 0.13 for the 1.3B model.<br><br>Table 2 compares GPT-Neo 2.7B performance under different training strategies. The Baseline model does not address subject-object co-occurrence biases, resulting in moderate Hits@1 scores. Undersampling removes biased samples, reducing training data and diversity. This increases overfitting risk and lowers generalization, especially in Gold Objects and Relation-wise evaluations. In contrast, MAGNET generates counterfactual samples that negate frequent object associations while preserving subjects. Learning from both original and counterfactual data maintains diversity and improves generalization, yielding substantially higher Hits@1 scores across all evaluation scenarios.<br><br>**Correlation Analysis**<br>We analyzed co-occurrence statistics in the Pile dataset [48], a pre-training dataset for GPT-Neo, and correlated them with LLMs' ability to probe factual knowledge. Entities with uncountable co-occurrence counts or consisting of more than three tokens (less than 6% of all entities) were excluded. We then computed correlations for (1) zero-shot, (2) fine-tuning alone, and (3) fine-tuning using MAGNET.<br><br>Fig 6 illustrates the number of samples in each joint subject-object frequency bin, organized according to the subject frequency bin.<br><br>Fig 7 shows co-occurrence correlations in the zero-shot setting. Hits@1 scores increase linearly with subject frequency up to approximately 10^4-10^5 for the joint subject-object frequency. However, for high-frequency subjects with relatively rare object occurrences, Hits@1 drops sharply. This indicates that LLMs struggle to predict rare facts due to co-occurrence bias.<br><br>Fig 8 presents co-occurrence correlations for fine-tuning and MAGNET. Fine-tuning roughly doubles Hits@1 compared to zero-shot but still shows sharp drops for rare facts. MAGNET, in contrast, improves overall performance by approximately three times over zero-shot and shows a slower performance decline, even for rare subject-object pairs. For bins with subject frequency 10^3-10^4 and joint frequency 10^1-10^2, MAGNET demonstrates about threefold robustness to co-occurrence bias compared to zero-shot and slower decline than standard fine-tuning.<br><br>Without MAGNET, GPT-Neo 2.7B produced 5,154 correct and 3,670 incorrect answers out of 8,824. With MAGNET, the model achieved 6,177 correct and 2,647 incorrect answers. This means 1,521 predictions changed from incorrect to correct, while 498 changed from correct to incorrect.<br><br>Among the 3,670 errors without MAGNET, 989 cases involved predictions of words with higher co-occurrence counts than the ground truth. MAGNET corrected 311 of these bias-induced errors (examples in Table 3). Conversely, of the 498 predictions that changed from correct to incorrect, 382 had higher conditional probabilities under the base model, indicating that MAGNET occasionally flipped answers despite the model's original preference for the correct option (examples in Table 4).<br><br>Overall, MAGNET effectively corrects bias-induced errors, though it occasionally flips correct answers to incorrect ones. These cases typically occur when the object distribution for a subject is relatively uniform, meaning no single object dominates co-occurrence statistics. As a future direction, constraining counterfactual generation to subjects with strongly skewed object distributions could reduce unnecessary flips and further improve model performance.<br><br>**Results for Open LLM**<br><br>We evaluated the impact of MAGNET on the target models across multiple benchmarks. In addition to TruthfulQA, we included HellaSwag and Winogrande, with results summarized in Table 5. For TruthfulQA, we used MC2 (Multi-true), which computes the normalized probability assigned to the correct answer set given multiple true/false options. HellaSwag and Winogrande were evaluated using multiple-choice accuracy, representing the proportion of correct selections among four candidate continuations and pronoun disambiguation questions, respectively. |

<table>
<tr><td></td><td>Models were trained on 4 RTX 3090 GPUs for 3 epochs, using a batch size of 256 and a learning rate of 2e-5. The Adam optimizer was employed with beta_1 = 0.9 and beta_2 = 0.999. All other procedures follow HuggingFace's causal language modeling scripts [58]. Details of fine-tuning and evaluation are provided in S3 File.<br><br>We further investigated the effect of training data size on GPT-Neo 125M using MAGNET, as shown in Fig 9. These experiments were single runs.<br><br>Overall, MAGNET effectively mitigates co-occurrence bias, reducing the likelihood of generating incorrect words with high co-occurrence probability. This improves the factual accuracy and truthfulness of model outputs.<br><br>Ablation Study<br><br>To evaluate the quality of counterfactual sentences, we conducted generation experiments using 1-shot, 5-shot, and 10-shot prompt settings. Results are summarized in Table 6.<br><br>As Table 6 shows, increasing the number of shots improves the model’s ability to follow the prompt and produce correctly formatted outputs, enabling more effective data collection. The 10-shot setting consistently yielded the highest performance across benchmarks.<br><br>To ensure the factual accuracy of generated sentences, we performed human filtering to verify that the subject and object were preserved. Table 7 compares performance with and without this filtering.<br><br>Human filtering consistently improved performance, confirming that maintaining subject-object fidelity and factual consistency enhances model outputs.<br><br>We also compared counterfactual generation using GPT-3.5 Turbo and GPT-4 Turbo. Table 8 summarizes the results.<br><br>As shown, GPT-4 Turbo consistently outperformed GPT-3.5 Turbo across all benchmarks. These results emphasize the importance of generating high-quality, factually grounded counterfactual sentences while preserving the subject-object structure. They also indicate that using a sufficient number of in-context shots and leveraging more advanced LLMs can further enhance MAGNET’s effectiveness, improving both the factual robustness and truthfulness of the target models.</td></tr>
<tr><td colspan="2">Reviewer #1 - Comment 2</td></tr>
<tr><td>Comment</td><td>Some figure captions (especially Figures 7–9) could be made more concise, and figure quality should be upgraded to ensure all text and visual elements are clearly visible.</td></tr>
<tr><td>Response</td><td>We thank the reviewer for the helpful suggestion regarding figure captions and quality. We have revised the captions for Figures 1, 2, 3, 4, 5, 7, 8, and 9 to make them more concise, and increased the text size in all figures to ensure that all labels and visual elements are clearly visible.<br><br>[Page 4](Figure 1) – Section Methods<br>[Page 5](Figure 2) – Section Methods<br>[Page 6](Figure 3) – Section Methods<br>[Page 6](Figure 4) – Section Results<br>[Page 7](Figure 5) – Section Results<br>[Page 8](Figure 7) – Section Results<br>[Page 8](Figure 8) – Section Results<br>[Page 9](Figure 9) – Section Results</td></tr>
<tr><td colspan="2">Reviewer #1 - Comment 3</td></tr>
<tr><td>Comment</td><td>Including a short summary table in the Methods section listing datasets, benchmarks, and evaluation metrics would improve readability.</td></tr>
<tr><td>Response</td><td>We thank the reviewer for this helpful suggestion. Because all datasets, benchmarks, and</td></tr>
</table>

| | |
|---|---|
| | evaluation metrics are introduced in the Results section, we added a summary table in that section along with a concise explanatory paragraph to improve readability and provide a clear overview of the experimental setup.<br><br>**[Page 6](Line 181–185) – Section Results**<br>**Results**<br><br>Table 1 provides a concise overview of the datasets, benchmarks, and evaluation metrics used in our experiments. Each experiment category—Factual Knowledge Probing, Counterfactual Training (MAGNET), Bias Analysis, and General Evaluation—is associated with its respective dataset and metrics, offering a clear summary of the experimental configuration prior to discussing detailed results.<br><br>**[Page 6](Table 1) – Section Results**<br>Overview of datasets, benchmarks, and corresponding evaluation metrics for each experiment category. |
| **Reviewer #1 - Comment 4** | |
| **Comment** | A light grammar and style edit is advised to smooth a few long sentences in the Introduction and Results sections. |
| **Response** | We appreciate the reviewer's suggestion regarding grammar and style. In response, we carefully revised both the Introduction and Results sections to smooth overly long sentences and improve clarity and readability throughout the manuscript.<br><br>**[Page 1-2](Line 2-30) – Section Introduction**<br>**Introduction**<br><br>Natural language processing (NLP) research has recently experienced rapid growth with the emergence of large language models (LLMs) [1,2]. LLMs have demonstrated strong performance across a wide range of NLP tasks, including natural language inference [3], question answering [4], common-sense reasoning [5], and translation [6]. They have also achieved significant gains in natural language generation tasks. However, the problem of hallucination—the generation of plausible but untruthful sentences—has attracted considerable attention. Early work focused on the likelihood-maximizing objective function used during training and decoding, showing that natural language generation models can produce sentences that are plausible yet nonsensical or untruthful [7,8].<br><br>Recent studies suggest that LLMs often learn spurious features, which can lead to untruthful sentences [9]. Inspired by [10], we identify co-occurrence statistics in pre-trained sentences as a major contributor to these spurious features. Kang et al. proposed a fine-tuning method that removes biased samples from the dataset. While this approach mitigates hallucination caused by high co-occurrence statistics, it can hurt generalization due to the reduced data size.<br><br>In this paper, we propose MAGNET (Model-AGNostic coutErfacTual synthesis and adaptive fine-tuning), a framework designed to address bias in fine-tuning datasets by generating counterfactual samples for all instances, rather than removing biased samples. Counterfactual samples have been widely used in NLP to mitigate spurious features such as co-occurrence bias [11], and several studies have leveraged them for data augmentation [12-15]. Most methods generate counterfactuals by identifying and replacing terms that play a crucial role in a sentence's causality.<br><br>Using MAGNET presents two main challenges. First, generating counterfactuals to address subject-object co-occurrence bias requires extracting the subject and object, typically using part-of-speech (POS) tagging. In our approach, we directly utilize the subject and object information provided by LAMA-TREx. Second, counterfactual sentences should retain the subject while negating the object. This task requires broad knowledge and common-sense reasoning. To address this, we leverage GPT-3's powerful few-shot learning ability to generate counterfactual sentences effectively. |

| | |
|---|---|
| | **[Page 6-11](Line 181-297) – Section Results**<br>**Results**<br><br>Table 1 provides a concise overview of the datasets, benchmarks, and evaluation metrics used in our experiments. Each experiment category—Factual Knowledge Probing, Counterfactual Training (MAGNET), Bias Analysis, and General Evaluation—is associated with its respective dataset and metrics, offering a clear summary of the experimental configuration prior to discussing detailed results.<br><br>**Factual Knowledge Probing**<br><br>Fig 4 shows the results of the Factual Knowledge Probing experiment in the study by \cite{kang2023impact}, which investigates the factual knowledge of LLMs using the LAMA-TREx dataset. The sentences used for validation are represented as subject-relation-object triples and converted into natural language using a predefined template. For example, the triple `Texas'-'capital'-'Austin' is converted to "The capital of Texas is Austin." Each fact masks the object and is converted into a Cloze statement (e.g., "The capital of Texas is [MASK]").<br><br>We trained the model for 3 epochs on 4 RTX 3090 GPUs. The batch size per device was 32, giving a total batch size of 128. The learning rate was 2e-5, and the Adam optimizer was used with beta_1 = 0.9 and beta_2 = 0.999.<br><br>For fine-tuning, the input prompt follows the format "### Input:\n {X\} \n\ n### Response:", where X is a masked sentence. For instance, "Hydatius has the position of [MASK]." The model is supervised to predict "bishop," which is the expected answer. Details are provided in S2 File.<br><br>The factual knowledge dataset contains 20,587 samples. We used 10,294 original sentences and 10,294 counterfactual samples as random samples to train MAGNET. To evaluate the quality of counterfactuals, we computed Self-BLEU, which measures sentence similarity. The score of 0.4668 indicates moderate diversity, showing that the generated sentences are sufficiently varied while remaining natural and coherent. This balance is important for effective fine-tuning.<br><br>For evaluation, we used Hits@1. It is 1 if the correct answer is ranked first among predicted candidates, and 0 otherwise. Because LLMs are not specifically trained for factual knowledge probing, we tested three restricted output vocabularies: (1) remove stopwords, (2) gold objects, and (3) gold objects (relation-wise). The first excludes NLTK 3.8.1 stopwords. The second restricts candidates to gold objects in the entire dataset, while the third restricts them per relation.<br><br>Fig 5 shows Hits@1 under a zero-shot setting with limited candidate sets. MAGNET improves the score by ~0.12 for the largest model and by 0.13 for the 1.3B model.<br><br>Table 2 compares GPT-Neo 2.7B performance under different training strategies. The Baseline model does not address subject-object co-occurrence biases, resulting in moderate Hits@1 scores. Undersampling removes biased samples, reducing training data and diversity. This increases overfitting risk and lowers generalization, especially in Gold Objects and Relation-wise evaluations. In contrast, MAGNET generates counterfactual samples that negate frequent object associations while preserving subjects. Learning from both original and counterfactual data maintains diversity and improves generalization, yielding substantially higher Hits@1 scores across all evaluation scenarios.<br><br>**Correlation Analysis**<br>We analyzed co-occurrence statistics in the Pile dataset [48], a pre-training dataset for GPT-Neo, and correlated them with LLMs' ability to probe factual knowledge. Entities with uncountable co-occurrence counts or consisting of more than three tokens (less than 6% of all entities) were excluded. We then computed correlations for (1) zero-shot, (2) fine-tuning alone, and (3) fine-tuning using MAGNET.<br><br>Fig 6 illustrates the number of samples in each joint subject-object frequency bin, organized according to the subject frequency bin. |

Fig 7 shows co-occurrence correlations in the zero-shot setting. Hits@1 scores increase linearly with subject frequency up to approximately 10^4-10^5 for the joint subject-object frequency. However, for high-frequency subjects with relatively rare object occurrences, Hits@1 drops sharply. This indicates that LLMs struggle to predict rare facts due to co-occurrence bias.

Fig 8 presents co-occurrence correlations for fine-tuning and MAGNET. Fine-tuning roughly doubles Hits@1 compared to zero-shot but still shows sharp drops for rare facts. MAGNET, in contrast, improves overall performance by approximately three times over zero-shot and shows a slower performance decline, even for rare subject-object pairs. For bins with subject frequency 10^3-10^4 and joint frequency 10^1-10^2, MAGNET demonstrates about threefold robustness to co-occurrence bias compared to zero-shot and slower decline than standard fine-tuning.

Without MAGNET, GPT-Neo 2.7B produced 5,154 correct and 3,670 incorrect answers out of 8,824. With MAGNET, the model achieved 6,177 correct and 2,647 incorrect answers. This means 1,521 predictions changed from incorrect to correct, while 498 changed from correct to incorrect.

Among the 3,670 errors without MAGNET, 989 cases involved predictions of words with higher co-occurrence counts than the ground truth. MAGNET corrected 311 of these bias-induced errors (examples in Table 3). Conversely, of the 498 predictions that changed from correct to incorrect, 382 had higher conditional probabilities under the base model, indicating that MAGNET occasionally flipped answers despite the model's original preference for the correct option (examples in Table 4).

Overall, MAGNET effectively corrects bias-induced errors, though it occasionally flips correct answers to incorrect ones. These cases typically occur when the object distribution for a subject is relatively uniform, meaning no single object dominates co-occurrence statistics. As a future direction, constraining counterfactual generation to subjects with strongly skewed object distributions could reduce unnecessary flips and further improve model performance.

**Results for Open LLM**

We evaluated the impact of MAGNET on the target models across multiple benchmarks. In addition to TruthfulQA, we included HellaSwag and Winogrande, with results summarized in Table 5. For TruthfulQA, we used MC2 (Multi-true), which computes the normalized probability assigned to the correct answer set given multiple true/false options. HellaSwag and Winogrande were evaluated using multiple-choice accuracy, representing the proportion of correct selections among four candidate continuations and pronoun disambiguation questions, respectively.

Models were trained on 4 RTX 3090 GPUs for 3 epochs, using a batch size of 256 and a learning rate of 2e-5. The Adam optimizer was employed with beta_1 = 0.9 and beta_2 = 0.999. All other procedures follow HuggingFace's causal language modeling scripts [58]. Details of fine-tuning and evaluation are provided in S3 File.

We further investigated the effect of training data size on GPT-Neo 125M using MAGNET, as shown in Fig 9. These experiments were single runs.

Overall, MAGNET effectively mitigates co-occurrence bias, reducing the likelihood of generating incorrect words with high co-occurrence probability. This improves the factual accuracy and truthfulness of model outputs.

**Ablation Study**

To evaluate the quality of counterfactual sentences, we conducted generation experiments using 1-shot, 5-shot, and 10-shot prompt settings. Results are summarized in Table 6.

As Table 6 shows, increasing the number of shots improves the model's ability to follow the prompt and produce correctly formatted outputs, enabling more effective data collection. The 10-

| | |
|---|---|
| | shot setting consistently yielded the highest performance across benchmarks.<br><br>To ensure the factual accuracy of generated sentences, we performed human filtering to verify that the subject and object were preserved. Table 7 compares performance with and without this filtering.<br><br>Human filtering consistently improved performance, confirming that maintaining subject-object fidelity and factual consistency enhances model outputs.<br><br>We also compared counterfactual generation using GPT-3.5 Turbo and GPT-4 Turbo. Table 8 summarizes the results.<br><br>As shown, GPT-4 Turbo consistently outperformed GPT-3.5 Turbo across all benchmarks. These results emphasize the importance of generating high-quality, factually grounded counterfactual sentences while preserving the subject-object structure. They also indicate that using a sufficient number of in-context shots and leveraging more advanced LLMs can further enhance MAGNET's effectiveness, improving both the factual robustness and truthfulness of the target models. |
| **Reviewer #1 - Comment 5** | |
| **Comment** | Verify reference formatting and ensure that all appendices (S1–S3) are correctly labeled and cited in the main text. |
| **Response** | We thank the reviewer for the valuable comment regarding reference formatting and appendix labeling. We have updated all references to their most recent officially published versions and unified the appendix labels by renaming S1–S3 as "Files" for consistency across the manuscript. In addition, we verified that File S1, File S2, and File S3 are correctly cited in the main text at lines 151, 200, and 272, respectively.<br><br>**[Page 12](Line 376–378) – Supporting information**<br><br>**S1 File. Prompts used to generate counterfactual samples.**<br>**S2 File. Data description of factual knowledge probing experiments.**<br>**S3 File. Description of datasets used with MAGNET for the target model.** |
